# Fragile-X Syndrome Is Associated With NMDA Receptor Hypofunction and Reduced Dendritic Complexity in Mature Dentate Granule Cells

**DOI:** 10.3389/fnmol.2018.00495

**Published:** 2019-01-17

**Authors:** Suk-Yu Yau, Luis Bettio, Jason Chiu, Christine Chiu, Brian R. Christie

**Affiliations:** Division of Medical Sciences, Island Medical Program, University of Victoria, Victoria, BC, Canada

**Keywords:** fragile X syndrome (FXS), NMDA (*N*-methy-D-aspartate receptor), dendrite complexity, neurogenesis, dentate gyrus, AMPA (α-amino-3-hydroxy-5-methyl-4-isoxazolepropionic acid)

## Abstract

Fragile X syndrome (FXS) is the most common form of inherited intellectual disability. It is caused by the overexpansion of cytosine-guanine-guanine (CGG) trinucleotide in *Fmr1* gene, resulting in complete loss of the fragile X mental retardation protein (FMRP). Previous studies using *Fmr1* knockout (*Fmr1* KO) mice have suggested that a *N*-methyl-D-aspartate receptors (NMDAR) hypofunction in the hippocampal dentate gyrus may partly contribute to cognitive impairments in FXS. Since activation of NMDAR plays an important role in dendritic arborization during neuronal development, we examined whether deficits in NMDAR function are associated with alterations in dendritic complexity in the hippocampal dentate region. The dentate granule cell layer (GCL) presents active postnatal neurogenesis, and consists of a heterogenous neuronal population with gradient ages from the superficial to its deep layer. Here, we show that neurons with multiple primary dendrites that reside in the outer GCL of *Fmr1* KO mice display significantly smaller NMDAR excitatory post-synaptic currents (EPSCs) and a higher α-amino-3-hydroxy-5-methyl-4-isoxazolepropionic acid (AMPA) to NMDA ratio in comparison to their wild-type counterparts. These deficits were associated with a significant decrease in dendritic complexity, with both dendritic length and number of intersections being significantly reduced. In contrast, although neurons with a single primary dendrite resided in the inner GCL of *Fmr1* KO mice had a trend toward a reduction in NMDAR EPSCs and a higher AMPA/NMDA ratio, no alterations were found in dendritic complexity at this developmental stage. Our data indicate that the loss of FMRP causes NMDAR deficits and reduced dendritic complexity in granule neurons with multiple primary dendrites which are thought to be more mature in the GCL.

## Introduction

Cognitive impairment is a frequently reported issue for patients with fragile X syndrome (FXS), which is the most common form of inherited intellectual disability, and the leading single gene cause of autism spectrum disorder (Rousseau et al., 1991; Kabakus et al., 2006; Alanay et al., 2007). This syndrome is caused by the transcriptional silencing of the fragile X mental retardation 1 (*Fmr1*) gene, resulting in the loss or mutation of its product, fragile X mental retardation protein (FMRP), a RNA-binding protein that associates with polyribosomes and regulates translation (Brown et al., 2001; Zalfa et al., 2003). FMRP has been shown to repress the translation of several targets, including proteins critical for synaptic function (Zalfa et al., 2003; Darnell et al., 2011). Of particular interest for the current study is the identification of interaction partners that play roles in signaling pathways related to synaptic plasticity (Tcherkezian et al., 2010) and dendritic structure (Schenck et al., 2003; De Rubeis et al., 2013).

Using *Fmr1* knockout (*Fmr1* KO) mice, we and others have demonstrated that the cognitive impairments in FXS may be linked to a disruption in *N*-methyl-D-aspartate receptor (NMDAR)-dependent synaptic plasticity in the hippocampal dentate gyrus (DG) (Yun and Trommer, 2011; Eadie et al., 2012; Franklin et al., 2014; Bostrom et al., 2015, 2016). The NMDA receptor forms a heterotetramer between two obligatory GluN1 subunits and two GluN2 subunits. The GluN2 subunits are differentially expressed during development, with GluN2B subunits initially being more highly expressed than GluN2A subunits early in neuronal development (Wenzel et al., 1997). The impact of NMDAR on dendritic structure can also have functional implications, as neuronal models indicate that dendritic morphology can have a significant impact on neuronal firing patterns (Mainen and Sejnowski, 1996).

Activation of NMDARs is known to play an important role in dendritic arborization and spine morphogenesis during neuronal development (Tolias et al., 2005). NMDARs appear to contribute to spine and dendrite formation; however their exact role remains controversial. Using specific GluN2 antagonists in cultured cells, GluN2A subunits have been associated with dendritic arborization, while GluN2B subunits were associated with dendritic spine formation (Henle et al., 2012). In contrast, introducing the GluN2B subunits into ventral spinal neurons in culture enhanced dendritic arborization, an effect not observed with GluN2A subunits (Sepulveda et al., 2010). Recently we have shown that genetic deletion of the Glun2A subunit significantly decreases dendritic growth in maturing dentate granule cells (Kannangara et al., 2014), suggesting that NMDA hypofunction in the DG may affect dendritic arborization in this brain region that exhibits developmental regulated changes in neurogenic activity (Gil-Mohapel et al., 2013).

In the current study we sought to determine if the reduction in NMDAR function observed in *Fmr1* KO animals was associated with developmental deficits in dendritic arborization of neurons in the hippocampal dentate granule cell layer (GCL) (Cameron and Mckay, 2001). The adult-born neurons of the DG have been shown to express primarily GluN2B subunits early on (Spampanato et al., 2012), but are also known to undergo extensive dendritic arborization as they migrate into an already extensively populated GCL (van Praag et al., 2002). Indeed, dendritic arborization and cell body positioning have been used to identify young and old neurons in the DG (Wang et al., 2000; Eadie et al., 2005). Newly generated neurons tend to be preferentially located in the inner layer of the GCL (Overstreet et al., 2004; Espósito et al., 2005; Redila and Christie, 2006), whereas more mature granule cells appear to be located in the outer GCL (Wang et al., 2000; Overstreet et al., 2004; Redila and Christie, 2006). Combined morphological and electrophysiological analyses also indicate that neurons in the outer GCL are morphologically more complex and thus have a lower series resistance than neurons in the inner GCL (Wang et al., 2000; van Praag et al., 2002; Kannangara et al., 2014). Using the location of neurons in the GCL as a means to select neurons for whole-cell patch clamp analyses, we investigated how the loss of FMRP affects NMDAR function and dendritic arborization of both younger and more mature hippocampal DG neurons.

## Materials and Methods

### Animals

Adult male *Fmr1* KO mice with a C57BL/6 genetic background (Bakker et al., 1994) and their wild-type (WT) littermates at the age of 4- to 6-week month old were used for the experiments. All mice housed with food and water available *ad libitum* on a 12 h light/dark cycle. All experiments were performed in accordance with the guidelines set out by the Canadian Council on Animal Care and approved by the University of Victoria Animal Care Committee.

### Electrophysiology

#### Electrophysiological Preparation

Adult mice were anesthetized with isoflurane, their brains removed, and transverse hippocampal slices were prepared as previously described (Vasuta et al., 2007). Briefly, hippocampal slices (350 μm) were acquired using a Vibratome 1500 (Ted Pella, Inc., Redding, CA, United States). The brain was immersed in oxygenated (95% O_2_/5% CO_2_) artificial cerebrospinal fluid (ACSF) containing (in mM) 125 NaCl, 3 KCl, 1.25 NaHPO_4_, 25 NaHCO_3_, 1 CaCl_2_, 6 MgCl_2_, and 25 Glucose at 4°C. After sectioning, slices were transferred to a holding chamber containing warm (30°C) oxygenated normal ACSF (nACSF) consisting of (in mM) 125 NaCl, 2.5 KCl, 1.25 NaHPO_4_, 25 NaHCO_3_, 2 CaCl_2_, 1.3 MgCl_2_, and 10 dextrose for 1 h before being used for electrophysiological recordings.

#### Whole Cell Recording

Cells were patched using a borosilicate glass recording electrode (5–7 MΩ) and the formation of a gigaseal (2 GΩ) was required prior to break-in. Recordings with a series resistance higher than 30 MΩ or presenting a variation of more than 10% were excluded from the analyses. The intracellular solution consisted of (in mM) 20 KCl, 120 K-gluconate, 4 NaCl, 0.1 EGTA, 4 ATP, 0.3 GTP, 14 Phosphocreatine (Osmolarity 270 mOsm/kg, pH 7.2) when action potentials were measured in current clamp mode. To examine NMDA/AMPA receptor mediated excitatory post-synaptic currents (EPSCs) in Voltage-Clamp mode, the internal solution was composed of (in mM) 135 Cesium methanesulfonate, 8 NaCl, 10 HEPES, 2 ATP, 0.3 GTP, 7 Phosphocreatine, 10 QX-314 (Osmolarity 280 mOsm/kg, pH 7.3) and biocytin (0.2–0.4%). In all cells, Alexa Fluor 488 (40 mM) was included in the intracellular solution to assist with the visualization and classification of granule cells. EPSCs were evoked with bipolar stimulating electrodes placed in the medial perforant pathway and recorded using Axopatch 200B amplifier and pClamp10 software (Axon Instruments). AMPAR-mediated EPSCs were measured at a holding voltage of -70 mV, while NMDA EPSCs were measured by applying a +40 mV holding potential in the presence of picrotoxin (100 μM) in nACSF. Some granule cells located in the inner cell layer did not display NMDA receptor currents; however, only granules cells showing both NMDAR and AMPAR EPSCs were included in the analyses.

### Intracellular Filling and Immunostaining of Biocytin-Filled Cells

Cells were filled with Alexa Fluor 488 together with biocytin for immediate visualization with fluorescence microscopy (Olympus Fluoview 1000). After each recording, the electrode was quickly retracted from the cell to help maintain cell integrity for histology. Slices were then fixed with 4% paraformaldehyde and left overnight at 4°C. The following day they were washed with 0.01 M PBS repeatedly and then incubated in 3% H_2_O_2_ for 45 min to block any endogenous peroxidase activity. Slices were then washed with 0.01 M PBS, before being incubated in an avidin-biotinylated HRP complex (ABC) solution containing 0.1% Triton-X for 48 h at room temperature. Biocytin-filled cells were visualized using a diaminobenzidine (DAB; Sigma-Aldrich) solution. Slices were then mounted onto gelatin-coated glass slides and allowed to dry at room temperature for 2 days before being dehydrated in graded ethanol and cover-slipped with Permount.

### Co-labeling of Biocytin-Filled Cells With Neuronal Markers

Following antigen retrieval in citric acid buffer for 15 min (pH 6.8 at 60°C), brain slices were washed thoroughly with 0.01 M PBS and then incubated with primary antibodies: rabbit anti-doublecortin (Abcam, 1: 200, Cat No.: ab18723) or mouse anti-NeuN (Millipore, 1: 200, Cat No.: MAB377) at 4°C for 3 days. Brain slices were then washed with PBS and incubated in Streptavidin-conjugated with Cy3 (Sigma, 1: 400 Cat No.: 6402) and Alexa Fluor 488 conjugated donkey anti-rabbit or donkey anti-mouse IgG antibodies (Life Technologies, 1: 200, Cat No.: S-11223) for 4 h at room temperature. Following washes in PBS, slides were coverslipped using Fluoromount (Thermo Fisher Scientific).

### Selection and Classification of Granule Cells

We and others have previously shown that granule cells located in the outer GCL tend to have several primary dendrites and more dendritic branching, while granule cells located in the inner GCL are more likely to have only one primary dendrite and less dendritic branching (Desmond and Levy, 1985; Green and Juraska, 1985; Claiborne et al., 1990; Wang et al., 2000; Kannangara et al., 2014). In accordance with our prior work, young and mature dentate granule neurons were selected based on their position in the inner and outer GCL, as well as their morphology, respectively. These cells typically had either single (inner cells) or multiple (outer cells) primary dendrites extending from the cell body, as previously reported (Wang et al., 2000; Bartesaghi and Serrai, 2001; Kannangara et al., 2014; Yau et al., 2016b).

### Sholl Analysis of Dendritic Complexity

Only those granule cells that exhibited intact dendrites with no cut branches were used in these analyses. Dendritic tracing was performed using Neurolucida software (MBF Bioscience, Williston, VT, United States) with a 40X objective lens. Sholl analysis was used to measure dendritic lengthen and dendritic branching with a concentric 10-μm interval as previously reported (Kannangara et al., 2014; Yau et al., 2016b).

### Statistical Analysis

Repeated measure ANOVA for dendritic branching and dendritic length analysis were performed using SPSS 14.0 (SPSS, Inc., Chicago, IL, United States). In some instances, two group comparisons between WT and *Fmr1* KO were performed using Student’s *t*-test. Data are presented as mean ± SEM. Statistical significance was indicated by a probability (*P*) value less than 0.05.

## Results

### Morphological Difference of Biocytin-Filled Granule Cells Located in the Inner and Outer Granule Cell Layers

**FIGURE 1:**
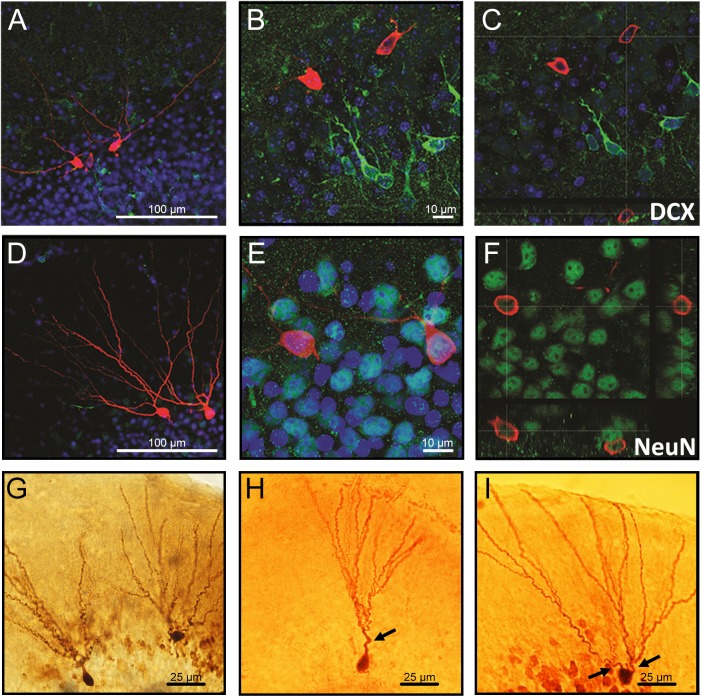
Confocal images of biocytin-filled cells in the granule cell layer (GCL) of the hippocampal dentate gyrus. **(A–C)** Biocytin-filled cells presenting multiple dendrites located in the outer GCL, while doublecortin-positive (DCX; immature neurons) cells located in the inner GCL layer. **(D–F)** Co-labeling of granule neurons projecting multiple dendrites from the soma with NeuN confirms that these cells are mature neurons. **(G)** Representative images of biocytin-filled neurons in the GCL of the hippocampal DG showing **(H)** an immature neuron localized in the inner layer and **(I)** a mature neuron localized in the outer cell layer. Red: biocytin-filled cells; Green: DCX **(A–C)** and NeuN **(D–F)**; Blue: Dapi nucleus staining; Arrows: primary dendrites. Slice thickness: 350 μm.

The neurons co-labeled with the immature neuronal marker doublecortin (DCX) displayed as the ones with a single primary dendrite were located in the inner cell layer (Figures 1A–C), while neurons co-labeled with the mature neuronal marker NeuN displayed multiple dendrites and were located in the outer cell layer (Figures 1D–F). Representative images showing differences in morphology and location of granule neurons in the GCL are demonstrated in Figures 1G–I.

### Action Potential and Membrane Properties of Dentate Granule Cells in *Fmr1* KO Mice

**FIGURE 2:**
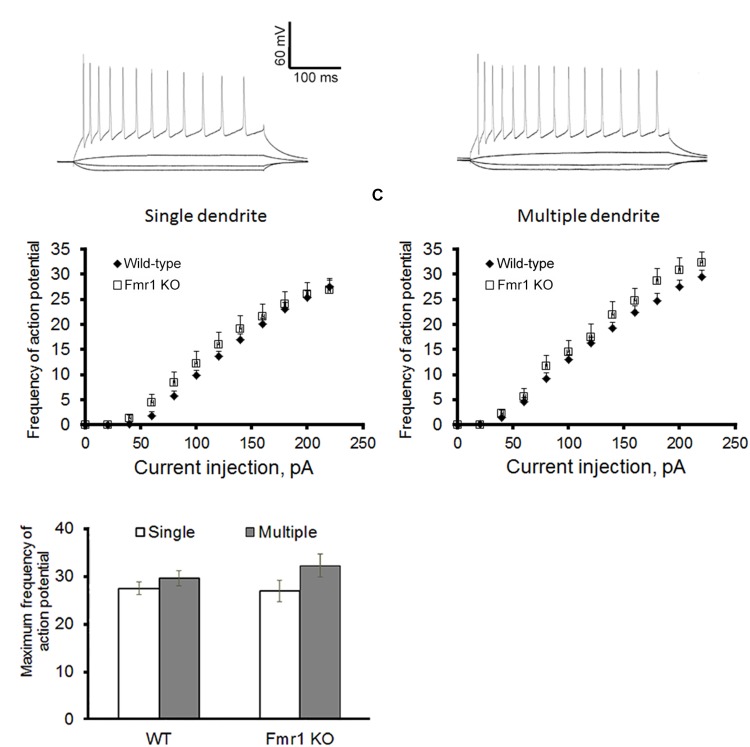
Action potential firing pattern of granule cells. **(A)** Representative traces of action potential train in neurons with single primary dendrite (left) or multiple primary dendrites (right) from WT mice. **(B)** Loss of FMRP did not affect firing pattern of granule neurons with single primary dendrite or **(C)** multiple primary dendrites. **(D)** However, neurons with multiple primary dendrites display a trend toward a higher maximum action potential firing rate when compared to the ones with a single primary dendrite. Neurons with single primary dendrite: WT: *n* = 12, *Fmr1* KO: *n* = 8; neurons with multiple primary dendrites: WT: *n* = 16, *Fmr1* KO: *n* = 8 (five mice per group).

Figure 2A shows a representation of action potential trains in neurons with a single primary dendrite or multiple primary dendrites from WT mice. There were no significant differences in action potential frequency when comparing granule cells from WT to *Fmr1* KO animals (single primary dendrite: *F*_1,20_ = 0.007, *P* = 0.933, Figure 2B; multiple primary dendrites: *F*_1,24_ = 1.158, *P* = 0.293, Figure 2C). However, granule neurons with multiple primary dendrites from *Fmr1* KO mice displayed a trend toward a higher maximum frequency of action potential (Student’s *t*-test, WT: *P* = 0.156; *Fmr1* KO: *P* = 0.05, Figure 2D), and a trend toward a lower input resistance when compared with the ones with a single primary dendrite (Table 1).

**Table 1:** Membrane properties of granule cells with single and multiple primary dendrites that display both AMPAR and NMDAR-EPSCs.

	WT		*Fmr1* KO		
	Single	Multiple	Single	Multiple	*P*-value
Ri (mΩ)	344.99 ± 15.1	223.45 ± 8.92	299.21 ± 32.4	211.09 ± 16.7	>0.05
RMP (mV)	-74.06 ± 1.03	-75.9 ± 0.60	-72.44 ± 3.73	-72.68 ± 1.12	>0.05


### Decreased NMDA EPSCs in Hippocampal Granule Cells From *Fmr1* KO Mice

**FIGURE 3:**
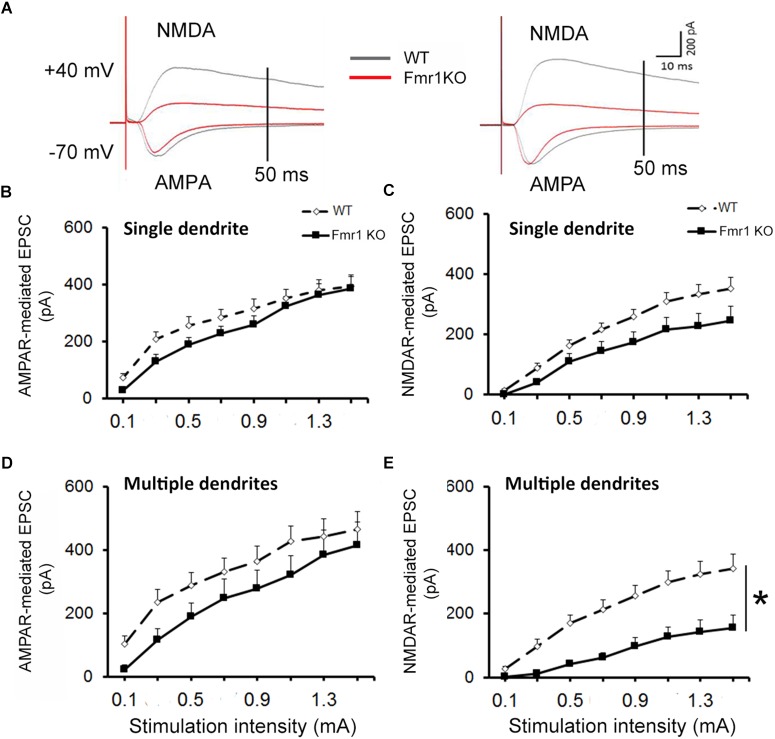
Decreased NMDAR-mediated EPSC in dentate granule cells of *Fmr1* KO mice. **(A)** Representative traces of granule neurons. Neurons with a single primary dendrite (left panel) and neurons with multiple dendrites (right panel) at maximal amplitude were shown. **(B)** No significant difference in AMPAR-mediated EPSC was observed between *Fmr1* KO and WT mice. **(C)** A trend toward a lower NMDAR-mediated EPSC was found in *Fmr1* KO mice when compared to WT mice (*P* = 0.067). **(D)** No significant difference in AMPAR-mediated EPSCs of older granule neurons was observed between *Fmr1* KO and WT mice; **(E)** but a significant decrease in NMDAR-mediated EPSC was observed in *Fmr1* KO mice when compared to WT mice. ^∗^*P* < 0.005 (*n* = 9–14; five mice per group; ANOVA, repeated measures).

For all recordings, AMPAR and NMDAR EPSCs were evoked using increasing stimulation intensity to construct I/O curves and determine the maximum response size. Representative traces acquired from granule neurons are depicted in Figure 3A. Similar levels of AMPAR-mediated EPSCs were recorded in younger granule cells from both experimental groups (*F*_1,25_ = 1.36, *P* = 0.254, Figure 3B), while a trend toward a reduction in NMDAR response size was observed in the *Fmr1* KO mice when compared to their WT littermates (*F*_1,25_ = 3.69, *P* = 0.067, Figure 3C). No alterations were found in AMPAR-mediated EPSCs of mature cells (*F*_1,20_ = 1.82, *P* = 0.194, Figure 3D), but a significant reduction in NMDAR-mediated EPSCs was observed in mature granule cells from *Fmr1* KO mice (*F*_1,20_ = 14.843, *P* = 0.001, Figure 3E).

**FIGURE 4:**
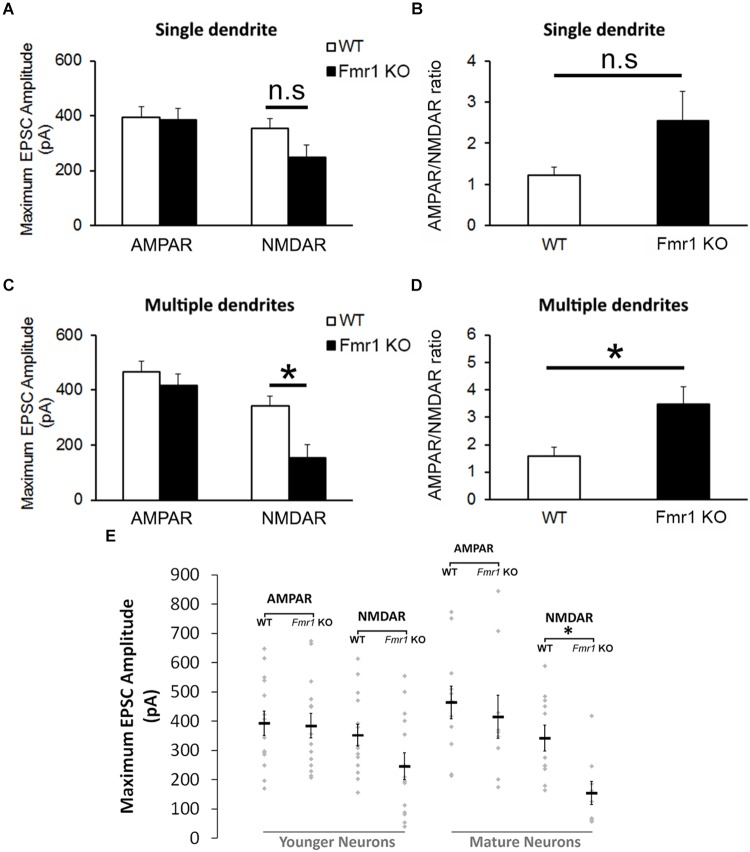
Altered NMDA currents and AMPA/NMDA ratio in *Fmr1* KO mice. **(A)** No difference was found in AMPAR-mediated EPSCs from neurons with a single primary dendrite, but a trend toward a reduction in NMDAR-mediated EPSCs (*P* = 0.09) and **(B)** an increase in AMPAR/NMDAR ratio (*P* = 0.07) were observed in *Fmr1* KO mice. **(C)** Although neurons with multiple primary dendrites from both groups demonstrated similar responses of AMPAR-mediated EPSCs, a significant decrease in NMDAR-mediated EPSCs **(D)** and a significant higher AMPAR/NMDAR ratio were found in *Fmr1* KO mice when compared to WT mice. **(E)** Scatterplot showing the amplitude of AMPAR- and NMDAR-mediated responses of individual neurons at distinct developmental stages in the hippocampal dentate gyrus of WT and *Fmr1* KO mice. ^∗^*P* < 0.05; n.s., non-significant (*n* = 9–14; five mice per group; Student’s *t*-test).

The analyses of the maximum amplitude of AMPAR-mediated EPSCs in neurons with a single primary dendrite did not reveal a significant difference between genotypes (*P* = 0.89), but a trend toward a reduction in NMDAR EPSCs was observed in cells from *Fmr1* KO mice in comparison with WT mice (*P* = 0.09, Figure 4A). This trend was also observed in the AMPAR/NMDAR ratio (*P* = 0.07, Figure 4B). A significant decrease in absolute maximum NMDAR-mediated EPSCs was found in mature neurons from *Fmr1* KO mice (*P* < 0.01, Figure 4C), as well as an increase in the AMPAR/NMDAR ratio (*P* < 0.05, Figure 4D) when compared to their WT counterparts. The amplitude of individual AMPAR- and NMDAR-mediated responses from hippocampal neurons at distinct developmental stages is depicted in Figure 4E.

### *Fmr1* KO Mice Present Decreased Dendritic Complexity in More Mature Granule Cells, but Not Younger Granule Cells

**FIGURE 5:**
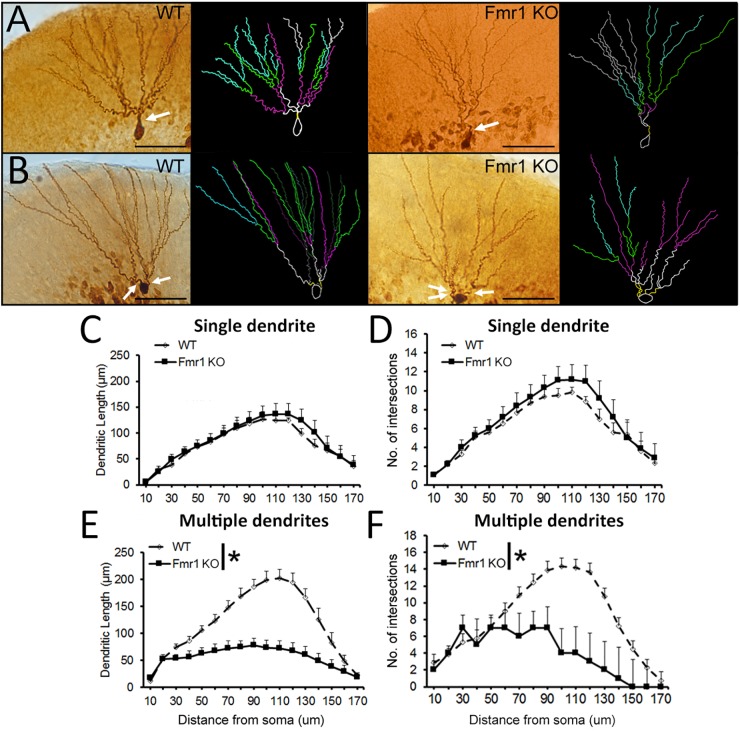
Sholl analysis of biocytin-filled granule cells. **(A)** Representative images of younger and **(B)** mature granule neurons from WT and *Fmr1* KO mice. Scale bar: 50 μm. **(C,D)** There were no differences in dendritic length and number of intersections in younger granule cells between WT (*n* = 10) and *Fmr1* KO mice (*n* = 8). **(E,F)** Dendritic length and number of intersections in more mature granule cells were significantly decreased in *Fmr1* KO mice (*n* = 10) when compared to WT mice (*n* = 12). Arrows: primary dendrites (yellow traces). ^∗^*P* < 0.005 (five animals per group; ANOVA, repeated measures).

Examples of biocytin filled cells used for the analysis of dendritic complexity are shown in Figures 5A,B. Sholl analysis revealed that WT and *Fmr1* KO mice presented similar dendritic measures (dendritic length: *F*_1,18_ **=** 0.185, *P* = 0.673, Figure 5C; dendritic branching: *F*_1,18_ = 0.473, *P* = 0.501, Figure 5D). Conversely, cells with multiple primary dendrites from *Fmr1* KO mice displayed a drastic reduction in both dendritic length (*F*_1,22_ **=** 26.291, *P* < 0.005, Figure 5E) and number of intersections (*F*_1,22_ = 15.301, *P* = 0.001, Figure 5F) when compared to their WT littermates. These findings indicate that the loss of FMRP may lead to a significant decrease in the development of dendritic complexity in more mature neurons.

## Discussion

The current study indicates that a loss of FMRP results in a significant reduction in NMDAR-mediated EPSCs and a concomitant decrease in dendritic complexity in hippocampal granule cells. Our findings also indicate that these deficits are primarily observed in the mature granule neurons which are with multiple primary dendritic processes arising from the soma, and are primarily located in the outer GCL. On the other hand, younger granule neurons that reside in the inner GCL, did not show a significant change in dendritic complexity, although they did show a trend toward a reduction in NMDAR EPSCs induced by the lack of FMRP. These data further support our previous findings indicating that *Fmr1* KO mice present a dysfunction in NMDAR in the hippocampal DG (Bostrom et al., 2015; Yau et al., 2016a), and show for the first time that these deficits are preferentially associated with a reduction in dendritic arborization in a specific population of granule cells in the outer GCL.

Dendritic morphology can affect synaptic plasticity by modulating neuronal firing rates, as well as altering the propagation of EPSPs and action potentials (Mainen and Sejnowski, 1996; Magee, 2000; Vetter et al., 2001). Loss of FMRP has been shown to reduce pruning of dendrites in the somatosensory cortex (Galvez et al., 2003) and in mitral cells of the olfactory bulb (Galvez et al., 2005), suggesting that this protein plays an important role in the development of dendritic processes. In the current study, we found that the loss of FMRP leads to a significant decrease in dendritic complexity in a specific subpopulation of granule neurons located in the outer GCL. This population of newborn neurons present multiple primary dendrites arising from the cell body and has been previously classified as being “mature neurons” based on morphological and electrophysiological analyses (Liu et al., 1996; Wang et al., 2000; Bartesaghi and Serrai, 2001; Kannangara et al., 2014). Our findings are in line with previous studies showing that the lack of FMRP leads to significant impairments in neurite extension of primary neural progenitor cells derived from the hippocampal DG (Guo et al., 2011). In addition, an *in vivo* study using retroviral labeling with green fluorescent protein found that mature newborn neurons from *Fmr1* KO mice present a decrease in dendritic complexity when compared to their WT counterparts (Guo et al., 2012). Our study corroborate these findings and provide further evidence indicating that the lack of FMRP affects hippocampal newborn cells in an age-dependent manner.

The regulation of neuronal morphology and synaptic modifications depends on the remodeling of the actin cytoskeleton, which in turn has been previously shown to be regulated by FMRP (Dictenberg et al., 2008; Nolze et al., 2013). Thus, it is possible that the deficits in dendritic length and branching observed in the present study are related to alterations in the organization of actin filaments induced by the lack of FMRP in the hippocampal DG. These changes may be related to the NMDAR hypofunction we observed, since these receptors are known to play a critical role in activity-dependent development of dendritic arbors and synaptogenesis (Nikonenko et al., 2002; Wong and Ghosh, 2002). It may be that the deficits in NMDAR function observed in our study in turn affect several NMDAR-dependent proteins who exert an influence on dendritic morphology. These may include extracellular signal-regulated kinases (Vaillant et al., 2002), Ca^2+^/calmodulin-dependent protein kinase II (Shi and Ethell, 2006), Rho family GTPases (Tolias et al., 2005) and glycogen synthase kinase 3 β (GSK-3β) (Rui et al., 2013). These proteins are interesting targets for future studies, particularly GSK-3β, since alterations in the translation of this protein seem to play a major role in the impaired differentiation of hippocampal neurons in FXS (Luo et al., 2010; Guo et al., 2012).

Prior research indicates that GluN2A subunit-containing NMDARs are important for dendritic arborization (Kannangara et al., 2014), while GluN2B may be more critical in regulating spine formation (Henle et al., 2012). Interestingly, we have previously shown that NMDAR hypofunction is associated with a significant decrease in the expression of both GluN2A and GluN2B subunits in the DG of *Fmr1* KO mice (Bostrom et al., 2015). However, because FMRP is a translational repressor, it is still not clear how its deletion leads to a reduction in the expression of NMDAR subunits, as well as to what extent it affects temporal changes in GluN2A and GluN2B expression in younger and more mature dentate granule cells. One factor that could be contributing to this deficit is the dysregulation of translational responses induced by excessive mGluR activation (Muddashetty et al., 2007), disruption of PSD-95 and CAMKII activities (Zalfa et al., 2007) and/or by the abnormal morphology of dendritic spines seen in FXS (Comery et al., 1997; Bilousova et al., 2009). On the other hand, since the loss of FMRP has an impact in the activity of a vast number of proteins, we cannot rule out the possibility that NMDAR-independent mechanisms are also contributing to the alterations in dendritic development found in our study. Regardless, the fact that we did not observe significant reductions in dendritic complexity and NMDAR function in the younger population of granule cells, suggests that the effects of FMRP on NMDAR function and dendritic complexity may occur over time. The DG offers a unique opportunity to study developmental changes, as it is one of the brain areas that exhibits continual neurogenesis throughout the lifespan. Indeed, the loss of FMRP also alters hippocampal neurogenesis in adult animals, which show increased cell proliferation, but impaired neuronal differentiation (Luo et al., 2010; Guo et al., 2012). Together these findings may suggest that the system is trying to compensate for reduced synaptic signaling by enhancing the production of new cells, but that loss of FMRP also negatively impacts the development of these cells. Our data indicate that a loss of FMRP causes significant deficits in both NMDAR function and dendritic arborization in mature neurons, and this could contribute to the abberant neurogenic process and reduced cognitive performance that has been observed with the loss of FMRP.

## Author Contributions

All authors made substantial contributions to the conception or design of the work; or the acquisition, analysis or interpretation of data for the work. Drafting the work or revising it critically for important intellectual content. Provide approval for publication of the content. Agree to be accountable for all aspects of the work in ensuring that questions related to the accuracy or integrity of any part of the work are appropriately investigated and resolved.

## Conflict of Interest Statement

The authors declare that the research was conducted in the absence of any commercial or financial relationships that could be construed as a potential conflict of interest.
